# The Paradox of Endometriosis in Mayer-Rokitansky-Kuster-Hauser Syndrome: Applying Three Criteria to Discriminate Between Retrograde Menstruation/Implantation and Coelomic Metaplasia/Embryonic Cell Rests Theories

**DOI:** 10.3390/jcm15041599

**Published:** 2026-02-19

**Authors:** Lutz Konrad, Muhammad Assad Riaz, Felix Zeppernick, Magdalena Zeppernick, Ivo Meinhold-Heerlein, Noemi Salmeri, Paola Viganò, Edgardo Somigliana, Paolo Vercellini

**Affiliations:** 1 Institute of Gynecology and Obstetrics, Faculty of Medicine, Justus Liebig University Giessen, Feulgenstr. 10-12, 35392 Giessen, Germany; muhammad.a.riaz@gyn.med.uni-giessen.de (M.A.R.); felix.zeppernick@gyn.med.uni-giessen.de (F.Z.); magdalena.zeppernick@gyn.med.uni-giessen.de (M.Z.); ivo.meinhold-heerlein@gyn.med.uni-giessen.de (I.M.-H.); 2 Academic Centre for Research on Adenomyosis and Endometriosis, Department of Clinical Sciences and Community Health, Università degli Studi di Milano, 20122 Milano, Italy; noemi.salmeri@unimi.it (N.S.); paola.vigano@policlinico.mi.it (P.V.); edgardo.somigliana@unimi.it (E.S.); paolo.vercellini@unimi.it (P.V.); 3 Department of Maternal and Child Health, Fondazione IRCCS Ca’ Granda Ospedale Maggiore Policlinico, Via Commenda 12, 20122 Milano, Italy

**Keywords:** endometriosis, endometrium, Mayer-Rokitansky-Kuster-Hauser syndrome, uterine remnants, retrograde menstruation, implantation, coelomic metaplasia, embryonic cell rests, theory, hypothesis

## Abstract

**Background/Objectives:** The scientific community is still divided between supporters of the implantation theory and researchers who advocate the theory of coelomic metaplasia/embryonic cell remnants to explain the initiation of endometriosis. A frequently cited argument in favor of the coelomic metaplasia/embryonic cell remnants theory is the occurrence of endometriosis in the Mayer-Rokitansky-Kuster-Hauser syndrome, since retrograde menstruation is not possible without endometrium. However, nearly all women with uterovaginal agenesis have uterine remnants that harbour islets of endometrium. **Methods:** To verify the validity of the coelomic metaplasia/embryonic cell rests theory, we analysed all reports of endometriosis in patients with Mayer-Rokitansky-Kuster-Hauser syndrome without endometrium, published between 1980 and 2025. Three criteria had to be met in order to clearly demonstrate the absence of endometrium and the presence of endometriosis: (i) preoperative imaging, (ii) surgical visualization, and (iii) histological examination. **Results:** None of the nine reports fully met all three criteria, and the presence of endometrium could never be ruled out. In addition, we used ten characteristics to assess the ‘goodness’ of a theory: testability, logical coherence, conceptual clarity and comprehensibility, external consistency, empirical validity, predictive power, parsimony, broad applicability, practical utility, and heuristic value. **Conclusions:** Overall, the implantation theory appears to fully satisfy all criteria to explain the onset of endometriosis in Mayer-Rokitansky-Kuster-Hauser syndrome. In contrast, the coelomic metaplasia/embryonic cell rests theory satisfies eight criteria only partly and does not satisfy two of them. Therefore, the null hypothesis that endometriosis can be present in the absence of endometrium in patients with utero-vaginal agenesis can be reasonably rejected.

## 1. Introduction

The primary cause of endometriosis *initiation* is still debated [1,2,3,4,5,6,7,8,9,10,11,12,13,14,15,16], and the Endometriosis Initiative Group [17] indicated ‘etiology/pathogenesis’ as a research priority. Endometriosis is likely a multifactorial disease, with many factors acting alone or in combination to influence whether early ectopic endometrial lesions progress to advanced forms of the disease [18,19,20,21]. Regardless of the contributing secondary factors [22], without the first step, the primary factor, the initial appearance of endometrial cells in ectopic locations, disease promotion and progression would not be possible.

Numerous pieces of evidence support the assumption that endometrial cells implant in extra-uterine locations through retrograde menstruation (RM/I) and lymphatic and hematogenous spread, thereby causing endometriosis [23,24,25]. However, several researchers argue that the RM/I theory cannot be applied to cases of endometriosis in patients with Mayer-Rokitansky-Kuster-Hauser syndrome (MRKHS), as either the uterus is absent or only uterine remnants (UR) without functional endometrium (FE) are present [21,26,27,28]. These cases are often cited to support the hypotheses of coelomic metaplasia and embryonic cell remnants (CM/ECR) [29]. Since there is no definition of uterine remnants, we must rely on the judgment of the pathologist.

In 1925, Sampson addressed the possible dual origin of ectopic endometrial foci, regardless of whether they originate in the uterine cavity or arise from in situ ectopic development. He defined the latter group as “developmentally misplaced pseudoendometrial tissue arising from remnants of Wolffian bodies, metaplasia of the peritoneal serosa, and possibly other sources” [30]. According to the coelomic metaplasia model [31,32], mesothelial cells originating from the coelomic cavity retain the potential to differentiate into endometrial cells spontaneously or under the influence of inducing factors such as estrogen, which is synthesized after the onset of ovarian function (reviewed in [22]. Nisolle and Donnez [27] believe that the metaplasia hypothesis specifically explains the formation of endometriomas, which would develop as a result of invagination of the coelomic epithelium into the ovarian cortex. These researchers support their view with MRKHS patients, as they “*do not have a uterus and therefore do not experience retrograde menstruation*.”

According to CM/ECR theory, primordial germ cells can spread into the body cavity during the development of the Wolffian and Müllerian ducts and remain there until estradiol synthesis begins in the ovaries [33,34]. Ovulation-induced hyperestrogenism stimulates these dormant embryonic cells to begin differentiating into endometrial cell types. After menarche, this can lead to endometriosis, particularly in the posterior pelvic region along the migration pathway of the embryonic Müllerian system [28]. Thus, a common embryonic developmental disorder could be the origin of both uterine-vaginal aplasia and endometriosis. Nisolle and Donnez [27] suggest that rectovaginal endometriotic lesions arise from these embryonic remnants.

Patients with MRKH usually have normal ovarian function and a normal karyotype, but significant hypoplasia or complete absence of the vagina, cervix, and uterus is often observed in 1 of 5000 women [35]. In the vast majority of cases, primary amenorrhea with typical adrenarche and telarche, possibly associated with congenital urological and skeletal disorders, leads to the initial presentation. Preoperative radiological examinations (ultrasound and MRI) and laboratory tests (testosterone, follicle-stimulating hormone [FSH], and karyotype) are necessary to make the differential diagnosis between vaginal and uterine obstructions. Diagnostic laparoscopy is only necessary in cases of endometriosis [36]. Vaginal dilation is the first line of treatment with a very high success rate around 93%.

After a century of intense debate [36,37,38,39,40], one of the questions that still needs to be addressed in the immediate future is: “*Where do endometriosis cells originate?*” [17]. In this regard, the MRKHS could be considered an excellent in vivo pseudo-experimental model with which to explain the primary stage of the endometriosis trajectory: “*what is/are the cell(s) of origin of endometriosis?*” [17].

We therefore considered it appropriate to reevaluate MRKHS patients with endometriosis who, according to reports, did not have FE [41,42] based on defined criteria. The null hypothesis was that MRKHS patients without UR or UR/FE (MRKHSFE-) have endometriosis, as only these cases clearly support the CM/ECR theory. The information for this statement was obtained by updating in June 2025 the systematic search conducted in June 2024 (for details, see [43]). As an extension of the results of our critical analysis, we perform a theory-appraisal exercise of the RM/I and CM/ECR theories with regard to the MRKHS model.

## 2. Definition of the Three MRKHSFE-Criteria and Their Application to Reports of Endometriosis in MRKHS Patients Without FE

### 2.1. Definition of the Three MRKHS-Criteria

Konrad et al. [22] identified some key criteria that are essential for indisputably confirming the absence of UR or FE/UR in MRKHS patients with endometriosis. Modifying Konrad et al.’s original suggestions [22], we have defined and adopted the three following intentionally conservative criteria, preoperative imaging, surgical visualization and histological examination. While the three criteria are subjective, they are medically very well-founded. Firstly, an initial diagnosis should be made using MRI and/or ultrasound, which is standard practice. The second criterion concerns surgery, which is necessary in most cases to diagnose endometriosis. In this case, a biopsy should clarify whether remnants of the uterus could be responsible for the development of endometriosis. The pathological evaluation of biopsies for the diagnosis of endometriosis is standard practice in pathology. All criteria must be fully satisfied to unequivocally demonstrate the presence of endometriosis in any MRKHS patient despite the absence of FE. This would corroborate the CM/ECR theory and expose the current problems with the RM/I theory:

#### 2.1.1. Preoperative Imaging

Uterine rests were found in 48–99% of MRKHS patients [22,44,45]. Magnetic resonance imaging (MRI) and ultrasound (US) were used to determine the presence of UR preoperatively. MRI in particular showed excellent test accuracy with a correlation of approximately 80% to 95% with direct visualization during surgery [22,46] and a high positive predictive value in identifying FE within the UR. However, MRI failed to detect the endometrium in 15% of suspected MRKHSFE- cases when a biopsy of the UR was performed [46]. In various studies, FE was present in 40% to 100% of the UR [44,45,47,48].

Pelvic ultrasound (US) is less accurate than MRI in correctly defining the characteristics of UR in MRKHS patients. Cooper et al. [45] argue that US should primarily serve as a screening tool to identify a normal female internal genital apparatus and therefore rule out MRKHS. Nonetheless, transrectal US is less accurate than transvaginal US in the detection of UR in individuals with almost complete vaginal agenesis [49]. Although US and MRI are not sufficiently reliable when FE is not identified within UR [22], in line with a ‘conservative’ approach, we also accepted preoperative US to identify UR with or without FE when a preoperative MRI was not performed.

In addition, the overall accuracy of preoperative imaging is higher when ruling out the presence of UR than when ruling out the presence of FE within UR. Conversely, test accuracy is very high when a UR with FE is detected [22,46,50]. Even if the negative predictive value of ultrasound and MRI is not optimal, these procedures help to select the patient group in which FE or UR is most likely not present by excluding all MRKHSFE+ individuals. This is precisely the population that is of interest for testing the plausibility of the CM/ECR theory for determining the origin of endometriosis.

#### 2.1.2. Surgical Visualisation

Especially when preoperative imaging does not detect UR, surgical findings are particularly important and can confirm the absence of UR with a very high degree of certainty. However, the presence of adhesions or abnormal UR locations can limit the reliability of surgery [51,52]. Furthermore, although almost all endometriosis phenotypes can be detected or biopsied during surgery, only adequate Tru-Cut needle biopsies provide samples if the UR has not been removed. Therefore, this first imaging and second surgical criterion should be combined when UR is not visible or cannot be removed [29,53].

#### 2.1.3. Histological Examination

A thorough examination of the entire removed UR by serial sections is the only reliable method to rule out the presence of FE, defined by the appearance of stroma and glands. However, UR are not systematically removed even in symptomatic patients, especially if no FE was detected in preoperative imaging and intraoperative Tru-Cut core biopsies of the UR are rarely performed. In order to histologically diagnose endometriosis in a removed ovarian cyst or peritoneal lesion, endometrium-like epithelial or stromal cells must also be clearly identifiable, and tissue biomarkers should be used in cases of doubt [22]. However, we did not consider this histochemical examination to be absolutely necessary to fully meet this third criterion. Nevertheless, when microphotographs were included in the publications reviewed, an independent assessment was performed by two experienced gynecological pathologists to confirm the claimed diagnosis of endometriosis (see the Acknowledgement section).

**Figure 1:**
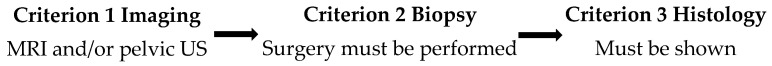
Flowchart of the three defined criteria to detect uteri remnants and endometriosis.

For the three criteria, we accepted MRI and US imaging, however, in cases of doubt we preferred MRI. Regarding surgery, the publication must state that it was performed to rule out the presence of uterine remnants and to confirm the diagnosis of endometriosis. In cases of discrepancies between imaging and surgery, we opted for surgery followed by histological evaluation, which ultimately determines whether a uterine remnant and endometriosis are present (Figure 1). Histological images must be presented; indications of their presence were considered insufficient.

We accepted both MRI and/or US, but surgery and histology of the uteri remnants and endometriosis lesions must be performed and shown, respectively. In cases of doubt, the histology is the deciding criterium.

### 2.2. Application of the Three MRKHSFE-Criteria

The search conducted to identify all cases of endometriosis in MRKHS patients without UR or FE/UR published between January 1980 and June 2025 yielded 9 reports. All articles in which endometriosis was found in MRKHS patients with FE (MRKHSFE+) were excluded, as most UR have normal fallopian tubes [45] and therefore RM is likely. Articles describing adenomyosis within the UR but without endometriosis were also excluded.

The 9 selected reports were assessed independently by two authors (P.V. and N.S.) and then verified by a third author (L.K.). The following three criteria were used for assessment: (i) absence of FE or UR in preoperative MRI or pelvic ultrasound if MRI was not available; (ii) a surgical description of the abdominal-pelvic anatomy with unobstructed visualization demonstrating the absence or presence of UR and the presence of endometriotic lesions. Preoperative imaging results and surgical findings were carefully compared, and discrepancies were noted. In the case of bilateral UR, there should be no major macroscopic asymmetry suggesting hematometra, especially if endometriotic lesions were present on the same side of the pelvis as the largest UR. (iii) A histological description of the endometriotic lesions biopsied during surgery or otherwise had to be available, with clear evidence of endometrium-like epithelium and/or stroma. The mere presence of iron-containing macrophages was not considered diagnostic for endometriosis. Published microphotographs were independently judged by two pathologists specializing in gynecological diseases and verified by the adjudicating overseer, as ‘fully satisfied’, ‘partly satisfied’, or ‘not satisfied’. According to our pre-planned analytical framework, all three criteria must be fully satisfied to validate the CM/ECR theory and falsify the RM/I theory.

The first identified report was published in 1981 [54] and the most recent in 2014 [55] (Table 1). The median age of the patients was 21.5 years [interquartile range, 15.5–23.5]. In one case, the age was not indicated [56]. None of the 9 reports considered fully satisfied all the three defined criteria (Table 1) which was the main objective of our analysis.

Only one report fully satisfied two criteria [55], and three reports fully satisfied a single criterion [58,59,66]. Five reports satisfied at least one criterion partly [54,56,57,60,67].

## 3. Conceptual Analysis to Explain the Onset of Endometriosis in Mrkhs Patients

In medicine, theories constitute basic, and sometimes partial, elucidations of complex biological phenomena. Consequently, theories, like explanations, may be good or poor. The MRKHS appears an ideal condition to test the two most popular explanations of how the endometrium develops in the pelvis, because it is a relatively easily controllable and circumscribed biological model. The primary goal was not to prove impossibility, but to test whether any convincing counterexample exist.

Several criteria have been recommended to assess the merit of theories in various fields of human research. We selected ten common criteria with the intention of comparing the ‘goodness’ of the RM/I and CM/ECR theories in explaining which is the *primum movens* of endometriosis onset in MRKHS patients, when the intrauterine endometrium is supposed to be lacking (Table 2).

The criteria were selected arbitrarily, but they include the ‘prerequisites for a good theory’ indicated by the Endometriosis Initiative Group [17], as well as several important, universally recognised principles that define the characteristics of any theory in all research fields (Table 2).

1. Testability (and falsifiability): A good theory can be tested through observation and experimentation to either confirm or disprove the theory. In the latter case, the theory is ‘falsified’ and it should not be repeatedly ‘falsified’; but rather discarded, and alternative explanations considered. If we assume that metaplasia could be a cause of endometriosis, we must clarify the question to what extent superficial peritoneal cells can transform into both endometrial epithelial and stromal cells. As an example, let us take ovarian surface epithelial cells (OSE), on which most experiments have been conducted. On the one hand, these cells are not purely stromal/mesenchymal cells, but on the other hand, they also have some epithelial characteristics [68,69], thus OSE is not firmly determined as most other adult epithelia [69]. Of note, OSE in ovarian inclusion cysts express epithelial genes more strongly and stromal/mesenchymal cells more weakly. Strictly speaking, this is not metaplasia, but rather a shift to an epithelial cell type originating from a mixed stromal/epithelial cell type. But more importantly, in 10 published cases involving 92 inclusion cysts, no woman developed endometriosis in the ovary [69]. In the case of endometriosis in MRKHS, the RM/I theory has been tested but not falsified, whereas the CM/ECR theory still has to proven.
2. Logical coherence (internal consistency): The various elements of the theory (assumptions, constructs, conditions, etc.) should be logically consistent with each other without contradictions. When endometriosis is observed in MRKHS patients, the RM/I theory does not violate logical rules. In contrast, the CM/ECR theory appears more elaborate and do not always easily fit within the overall construct. For example, the published data do not demonstrate the transition from mesothelial cells to epithelial and stromal endometrial cells [22] and as shown above inclusion cysts have never developed into endometrial glands [69].
3. Conceptual clarity and comprehensibility: A good theory is easy to understand, which is the case for the RM/I theory, whereas the CM/ECR theory requires an adequate knowledge of embryogenesis to be understood by professionals.
4. External consistency: A good theory should align with observations without contradicting facts. In the case of MRKHS, the RM/I theory aligns with current knowledge of endometriosis pathogenesis. The CM/ECR theory does not appear to reach the same degree of external consistency, as it does not align with what is known to be true in patients with obstructed menstruation in general and with obstructive Müllerian anomalies in particular [70].
5. Empirical validity: In MRKHS patients, the RM/I theory is supported by much more robust empirical evidence than the CM/ECR theory. For example, the proportion of MRKHSFE+ patients with endometriosis is significantly higher than the proportion in MRKHFE- patients, if there are any at all [22,43].
6. Predictive power: In MRKHS patients, the RM/I theory accurately predicts the development of endometriosis, especially in cases where FE is present within the UR. Conversely, the CM/ECR theory should have predicted the development of endometriosis in patients without FE within UR or without UR. However, based on a detailed analysis of the available data, this is not the case (Table 1).
7. Parsimony: A robust theory relies on fewer variables and makes fewer assumptions to explain several phenomena than more complex alternative theories. Thus, the simpler of the theories is more likely to be correct, a philosophical principle known as ‘Ockham’s Razor’, developed by the 14th-century English monk, theologian and logician, Father William of Ockham. In MRKHS patients, the RM/I theory is undoubtedly simpler than the CM/ECR theory [22], and according to the parsimony criterion, is more likely to be true.
8. Broad applicability (generalisability): A good theory should be comprehensive enough to explain a wide array of phenomena, can be generalised and applied to different populations and conditions, rather than being limited to specific cases. In this regard, the RM/I theory can be generalised also to populations without MRKHS, whereas the CM/ECR theory is restricted to cases with MRKHS and maybe ovarian endometriosis, although as argued above no definitive proof has ever been provided.
9. Practical utility: A good theory should have direct applications and contribute to solving real problems. The practical utility of the RM/I theory is easily comprehensible and applicable to MRKHS patients. When the main complaint is colicky and cyclical pelvic pain, the presence of FE must be thoroughly investigated and, if detected, the UR must be removed to avoid cryptomenorrhoea and solve the problem [45,70,71,72,73,74,75]. The CM/ECR theory focuses on treatment of endometriosis, but it risks overlooking the source of endometrial cells, thereby exposing to recurrence.
10. Heuristic value: A good theory should generate new hypotheses and stimulate empirical research, leading to further innovations. The RM/I theory appears generative because, if confirmed in the MRKHS model, it could form the basis of new secondary prevention strategies aimed at suppressing repetitive ovulatory menstruation in young women with severe symptoms. These approaches could be applied beyond the highly selective group of individuals with uterovaginal agenesis [18]. If a theory’s ability to generate new ideas and research were judged based exclusively on the number of reports including original data, RM/I would vastly outperform CM/ECR.

Overall, the RM/I theory appears to fully satisfy all the defined criteria for a “good theory” to explain the onset of endometriosis in MRKHS patients. Conversely, the CM/ECR theory only partly meets eight criteria and fails to satisfy two of them (Table 2). Although it is often claimed that peritoneal stem cells, endometrial stem cells or bone marrow cells can undergo metaplastic changes under the influence of hormonal or immune factors to transform into endometriotic cells to contribute to endometriosis [76], a definitive proof is still lacking. Although we cannot address all counterarguments here, it remains unclear how peritoneal stem cells develop into endometrial lesions (epithelial cells and stromal cells) at different locations in always exactly the same manner [22].

**Figure 2:**
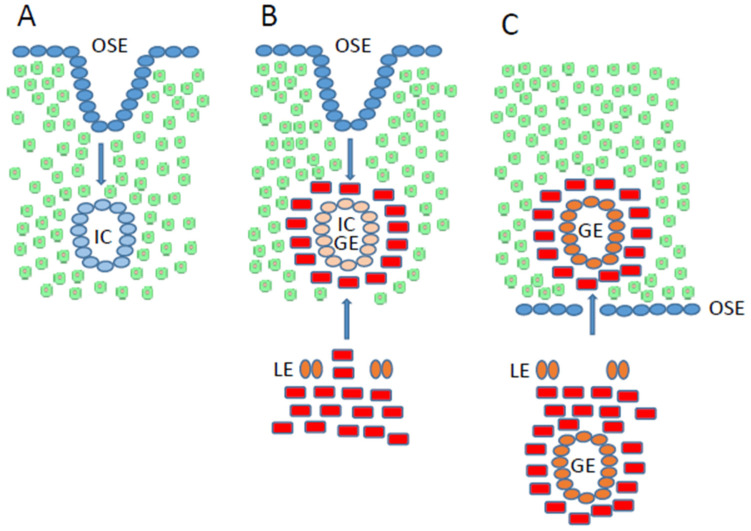
Model for inclusion cysts and ovarian endometriosis. (**A**) Inclusion cysts (IC) are caused by invagination of the ovarian surface epithelium (OSE, blue). The OSE in the inclusion cysts gains epithelial markers through metaplasia, but loses mesenchymal markers [69]. (**B**) It is assumed that after invagination, the OSE not only undergoes metaplastic changes but also forms endometrial glands (IC/GE) together with endometrial stromal cells (red) [77]. However, in cases of MRKHS without endometrium, the source of the endometrial stromal cells is unclear. The metaplasia of ovarian stromal cells (green) to endometrial stromal (red) cells has not yet been proven [22]. (**C**) The implantation hypothesis suggests that glands (GE) together with endometrial stromal cells invade the ovary and form ectopic lesions. LE, luminal epithelium; OSE, ovarian surface epithelium; GE, glandular epithelium; IC, inclusion cyst.

In summary, it can be stated that inclusion cysts arise from a metaplasia-like process of OSE (Figure 2A), but endometriosis clearly does not develop. In contrast, it has been claimed that inclusion cysts transform into ovarian endometriomas with or without endometrial stromal cells (Figure 2B) [77]. We already contradicted this publication in 2019 and pointed out that, in MRKHS without endometrium, this would require metaplasia of the OSE into endometrial stromal cells or conversion of the ovarian stromal cells into endometrial stromal cells [22], but to date, this evidence is still lacking. We therefore continue to support Sampson’s implantation theory (Figure 2C).

It has also been argued that extragenital endometriosis arises at least partially from metaplasia [15]. In two review articles, we were able to show that the implantation theory can indeed explain these manifestations as well [78]. Especially the laterality of endometriosis cannot be explained satisfactorily by the metaplasia theory [79,80].

## 4. Conclusions

We have tried to appraise the data on the origin of endometriosis in MRKHS patients as objectively as we were able to. However, to contextualise the arguments presented in this article, readers should be aware that the authors are staunch advocates of the RM/I theory and have published some reports and reviews on this topic [25,43,70,81,82,83]. This greatly increases the risk of confirmation bias due to our pre-existing beliefs. Therefore, we cannot exclude the possibility that we selectively searched for information supporting our preferred theory while disregarding evidence against it, belittling proofs against RM/I or interpreting them as consistent with or in favour of it.

Within the above scenario and based on the limited and poor-quality published evidence, the CM/ECR theory could not be corroborated in the cases of endometriosis observed in MRKHS patients purportedly without FE. None of the nine selected reports satisfied all three predefined criteria deemed mandatory for definitively diagnosing both, the presence of endometriosis and the absence of FE. Contrariwise, the RM/I theory withstood our critical analysis of the available data on this topic, emerging strengthened overall. Therefore, although both theories were demonstrated to be testable, the CM/ECR theory was shown to be highly unlikely, whereas the RM/I theory was not. Had the CM/ECR theory not been not been shown to be highly unlikely under the highly specific conditions of the MRKHS in vivo pseudo-experiment, the RM/I theory would have been severely weakened to the point that its validity would also have been called into question in all women without utero-vaginal agenesis.

Based on the present critical analysis, the RM/I theory can be defined as “good”, because it is clear, understandable, logical, consistent, undisputably parsimonious, testable, empirically valid, and predictive. Its broad applicability and practical utility could therefore be expressed through its heuristic value. Indeed, the RM/I theory has already generated novel research and stimulated the formulation of hypotheses [18].

We suggest that future studies should focus on establishing MRKH patient registries, standardizing diagnostic protocols, and conducting molecular analyses of lesions to clarify the origin of endometriosis in these cases. In conclusion, we have not found any prove without any doubt that endometriosis can be present in MRKHS patients without FE. Therefore, the long-held belief that ‘*endometriosis comes from the endometrium*’ [84] appears to still stand the test of time.

## Figures and Tables

**Table 1:** Assessment of MRKHS patients reporting the combination of (i) presence of endometriosis and (ii) absence of UR or FE/UR. Three criteria were used to validate or reject the ER/SM and/or RM/I hypothesis. Literature data are from 1980 until 2025.

Ref., Country, Patient Age	Criterion 1—Preoperative Imaging	Criterion 2—Surgical Visualisation	Criterion 3—Histological Examination	Authors Conclusions	Comment
[54], U.S.A., 23-yrs-old	– Absence of UR or FE/UR was not reported in the US.	± OMA in the left ovary. Two years later, two OMAs were removed.	– No histology and photomicrographs were provided.	“…*it is unlikely that tubal regurgitation is the only cause of endometriosis*”.	No demonstration is provided of the absence of FE/UR, as UR were neither biopsied nor removed.
[56], Turkey, age n.r.	± Absence of UR at US. No MRI.	± No surgical description is reported. Stage I endometriosis was detected.	– No histology and photomicrographs were provided.	“*One patient…without a uterus…, but with histologically proven endometriosis showed that this is not a universally valid dictum*”	Imaging, anatomical, and histopathology details are either lacking or insufficient to validate the authors’ conclusion.
[57], Turkey, 14-yrs-old	± Transabdominal sonogram showed an ovarian mass on the left side, and no UR was found. No MRI.	± A left OMA was histologically confirmed. No UR and ‘…*only remnants of rudimentary fallopian tubes*’ were described.	+/– Endometrial gland and stroma with hemosiderin laden macrophages in the left ovarian cysts were described, but photomicrographs were not provided.	*‘…müllerian-derived metaplasia in the ovaries…The beginning of ovulation might also trigger the metaplasia to end with endometrioma formation.’*	The authors state ‘*In our patient, menstruation was probably not possible due to the hypoplastic uterus and tubes*’. This indicates that at least an UR was present and thus absence cannot be ruled out, because of the missing MRI.
[58], Turkey, 17-yrs-old	+ Absence of uterus at preoperative US and MRI.	± Excision of a large right perirenal cyst. “…*uterus could not be detected and both tubes and the ovaries were normal*”.	± Pathology showed “*endometrial tissue and hemorrhage*” within the perirenal cyst. No histology and photomicrographs were provided.	The authors did not discuss whether their findings are in favour of the embryonic cell rests/coelomic metaplasia theory.	The inconsistent description of absence versus presence of the tubes at surgery, raises the doubt of misdiagnosis. Histological findings of endometriosis are provided.
[59], South Korea, 26-yrs-old	± Transrectal US showed no UR. No MRI.	+ A left OMA was excised. After adhesiolysis, no UR was found.	– Endometrial glands and stroma lining the cyst with hemosiderin-laden macrophages were identified. However, the photo-micrographs show hemosiderin-laden macrophages only.	‘…*we confirmed that there was no Müllerian structure in our patient. Our case cannot be adequately explained by Sampson’s theory of retrograde menstruation…Such conditions support the alternative theory of coelomic metaplasia*’	OMA was demonstrated in the absence of UR at both preoperative imaging and surgery. However, two expert pathologists and Konrad et al. [22] could not confirm the diagnosis of ovarian endometriotic cyst based on the low-quality photomicrographs (Figure 2a,b, page 995).
[60], U.S.A., 20-yrs-old and 25-yrs-old	± MRI confirmed vaginal and uterine agenesis, with possible presence of uterine horn remnants.	± Laparoscopies at the ages of 20 and 25 years detected no UR, and no fallopian tubes. Minimal superficial peritoneal endometriosis was observed on both occasions but destroyed using electrocautery.	– No histological confirmation of endometriosis is available as the superficial peritoneal implants were fulgurated without taking a biopsy.	*‘… our patient …did not develop pelvic pain until she was 20 years old. This 9-year timeperiod between onset of puberty… seems too long to justify the Müllerian rest theory, but it is quite sufficient to explain the development, and subsequent recurrence, of her endometriosis from transformation of totipotent cells’.*	MRI detected the ‘*possible presence of uterine horn remnants*’. Therefore, ‘*continuous oral contraceptive pills were initiated to minimize the risk of hematometra*’. Importantly, the reliability of the visual diagnosis of superficial peritoneal (stage I) endometriosis is variable and generally limited [61,62,63,64,65]. This case lacks histological confirmation of endometriosis.
[66], Canada; 12-yrs-old	± At MRI, the presence of *‘…functional endometrium in the setting of abnormal müllerian* *structures was suspected.’*	+ After a combined oral contraceptive continuously for 7 months, the right ovary/endometrioma and fallopian tube, midline UR and the bilateral fibrous bands with uterine horns were removed.	± *‘…a noncavitary uterine/Wolffian remnant lacking endometrium, a…fibrotic right ovary/endometrioma complex, and the right fallopian tube with a small paratubal cyst attached to a lateral uterine remnant without…discernible endometrium*.’ However, histological details and photomicrographs were not provided.	In the Abstract the authors state: *Patients with obstructive müllerian malformations with functional endometrium can be preoperatively managed with continuous combined low-dose monophasic oral contraceptives to control pain and treat endometriosis.*	*‘The pathologic findings…repre sent COC inhibition of functional endometrium in the right rudimentary uterine horn, allowing gradual resolution of the hematometra/endometriosis.’* Some inconsistencies between imaging, surgical, and histological findings do not allow definitive conclusions.
[67], China; 23-yrs-old	± A single uterine bud without FE was identified at transabdominal US. No MRI.	– No description of UR is provided.	– Endometrial glands or stromal cells of OMA cannot be identified in the poor-quality photo-micrograph (Figure 1; see comment)	At laparotomy, *‘*…a *purple and brown nodule measured 0.5 cm on the right ovary surface was… proved to be an ectopic endometriotic lesion. ’*	The absence of FE within UR is based on US only. The presence/absence of UR at surgery was not defined. No endometrial glands or stroma can be identified by two experts and Konrad et al. [22].
[55], Brazil; 24-yrs-old	+ Absent UR at MRI and US	± ‘*Two rudimentary uteruses and an endometrioma in the left ovary were observed*.’ The UR were not removed.	+ Pathology examination confirmed ovarian “*cystic endometriosis*”. However, histology and photomicrographs were not provided.	*‘*…*we present endometriosis in a patient with Mayer-Rokitansky-Küster-Hauser syndrome…uterus. This case reinforces the theory of coelomic metaplasia…rather than Sampson’s retrograde menstruation theory alone. ’*	Exclusion of FE within UR seems unreasonable. In fact, UR were observed at laparoscopy only, as preoperative US and MRI had incorrectly failed to detect them. However, absence of FE cannot be established at surgery/pathology, as the UR were not removed.

Ref., reference; yrs, years; n.r.; not reported; MRKHS = Mayer-Rokitansky-Kuster-Hauser syndrome; FE = functional endometrium; OMA = (ovarian) endometrioma; UR = uterine remnants; US = ultrasound; MRI = magnetic resonance imaging; COC = combined oral contraceptives. + = criterion fully satisfied; ± = criterion partly satisfied; – = criterion not satisfied.

**Table 2:** Ten features that should characterize a “good theory” for endometriosis initiation in MRKHS patients: Epistemological comparison between the retrograde menstruation/implantation theory (RM/I) and the coelomic metaplasia/embryonic cell rests (CM/ECR) theory *.

Characteristic	RM/I	CM/ECR
Testability (falsifiability)	+	±
Logical coherence (internal consistency)	+	±
Conceptual clarity and comprehensibility	+	±
External consistency	+	±
Empirical validity	+	±
Predictive power	+	–
Parsimony	+	–
Broad applicability (generalizability)	+	±
Practical utility	+	±
Heuristic value	+	±

* Mainly based on the principles indicated in the “Call for new theories” by The Endometriosis Initiative Group [17], and the findings of the systematic reviews by Konrad et al. [22] and Vercellini et al. [43]. + = criterion fully satisfied; ± = criterion partly satisfied; – = criterion not satisfied.

## Data Availability

The data included in this article were extracted as published in the available original articles. No new data were generated to support this paper. The data are available from the corresponding author upon request.
